# Inhibition of *Aspergillus fumigatus* and Its Biofilm by *Pseudomonas aeruginosa* Is Dependent on the Source, Phenotype and Growth Conditions of the Bacterium

**DOI:** 10.1371/journal.pone.0134692

**Published:** 2015-08-07

**Authors:** Jose A. G. Ferreira, John C. Penner, Richard B. Moss, Janus A. J. Haagensen, Karl V. Clemons, Alfred M. Spormann, Hasan Nazik, Kevin Cohen, Niaz Banaei, Elisabete Carolino, David A. Stevens

**Affiliations:** 1 California Institute for Medical Research, San Jose, California, United States of America; 2 Division of Infectious Diseases and Geographic Medicine, Department of Medicine, Stanford University, Stanford, California, United States of America; 3 Division of Pulmonology, Department of Pediatrics, Stanford University, Stanford, California, United States of America; 4 Department of Civil and Environmental Engineering, Stanford University, Stanford, California, United States of America; 5 Department of Medical Microbiology, Istanbul Faculty of Medicine, Istanbul University, Istanbul, Turkey; 6 Department of Pathology, Stanford University, Stanford, California, United States of America; 7 Escola Superior de Tecnologia da Saúde de Lisboa (Lisbon School of Health Technology), Lisbon, Portugal; Geisel School of Medicine at Dartmouth, UNITED STATES

## Abstract

*Aspergillus fumigatus (Af)* and *Pseudomonas aeruginosa* (*Pa*) are leading fungal and bacterial pathogens, respectively, in many clinical situations. Relevant to this, their interface and co-existence has been studied. In some experiments *in vitro*, *Pa* products have been defined that are inhibitory to *Af*. In some clinical situations, both can be biofilm producers, and biofilm could alter their physiology and affect their interaction. That may be most relevant to airways in cystic fibrosis (CF), where both are often prominent residents.

We have studied clinical *Pa* isolates from several sources for their effects on *Af*, including testing involving their biofilms. We show that the described inhibition of *Af* is related to the source and phenotype of the *Pa* isolate. *Pa* cells inhibited the growth and formation of *Af* biofilm from conidia, with CF isolates more inhibitory than non-CF isolates, and non-mucoid CF isolates most inhibitory. Inhibition did not require live *Pa* contact, as culture filtrates were also inhibitory, and again non-mucoid>mucoid CF>non-CF. Preformed *Af* biofilm was more resistant to *Pa*, and inhibition that occurred could be reproduced with filtrates. Inhibition of *Af* biofilm appears also dependent on bacterial growth conditions; filtrates from *Pa* grown as biofilm were more inhibitory than from *Pa* grown planktonically. The differences in *Pa* shown from these different sources are consistent with the extensive evolutionary *Pa* changes that have been described in association with chronic residence in CF airways, and may reflect adaptive changes to life in a polymicrobial environment.

## Introduction

Cystic fibrosis (CF) is the result of mutations in the CF transmembrane conductance regulator affecting epithelial chloride and bicarbonate transport. One result is development of thick respiratory secretions, which results in airway obstruction and recurrent episodes of lung inflammation and infection, leading to acute and chronic deterioration of lung function and a shortened lifespan [1]. The affected persons have defective mucociliary clearance and production of thick sticky mucus in which various pathogens can become entrapped. This is a suitable environment for microbial growth and colonization, and these organisms or their soluble metabolites contribute to airway inflammation and subsequent damage.

The most common bacterium and fungus infecting these airways are *Pseudomonas aeruginosa* (*Pa*) [2] and *Aspergillus fumigatus* (*Af*) [3–7], respectively, particularly in the chronically infected older patients. *Pa* evolves in CF airways, producing variants, such as those resulting in mucoid colony types, which are adapted to chronic residence there [2,8,9]. *Af* is ubiquitous in ambient air and the environment, and thus can be inhaled and subsequently establish residency. Both organisms are proficient adapters to environmental stress and relatively resistant to current antimicrobials. They are suspected as important agents in promoting mucus plug formation in the airways, and both are known to form biofilms in vitro and in vivo [2,9–20]. Microbes in biofilms have altered metabolism compared to the same organisms growing planktonically, and biofilms provide microbes with protection from host defenses as well as tolerance to some antimicrobial drugs [21]. The attribution of a role for these microbes in mucus plugging and biofilms stems from the known extracellular production of glycan polymers by *Af* [22] and alginate by *Pa* [2,9,17]. In addition to infection, *Af* can cause allergic bronchopulmonary aspergillosis in up to 15% of CF patients, a complication that causes repeated acute exacerbations, institution of immunosuppressive therapy, and accelerated decline in lung function [23]. As is the case with *Pa*, *Af* also produces secondary metabolites, in the environment as well as *in vivo*, which are known tissue toxins and also have immunosuppressive actions on the host response [24]. Infection with *Pa* or *Af* has been associated with a more rapid decline in CF pulmonary function [17,25–33], with the co-infected patients having the worst prognosis [32,34]. Both pathogens are also important because either can be an opportunist, causing invasive disease [35–39] or other complications [40,41], in lung transplantation, a therapeutic modality offered in debilitating CF.

It is therefore important to study the interactions between these two pathogens. *Pa*-secreted molecules have been well studied for their antifungal activities, a property that has been demonstrated in vitro [42–47]. These inhibitory molecules include homoserine lactones, pyocyanin and other phenazine derivates, pyrrolnitrin and fluorescent green pigments. However, these studies were performed with one or few *Pa* isolates, none representatives of variants that establish chronic residency in CF airways. Moreover, *Pa*-associated factors involved in this inter-kingdom inhibition continue to be elucidated [48]. The aim of this study was to evaluate the effect of different clinical *Pa* phenotypic variants obtained from CF and non-CF patients on *Af* biofilm formation and preformed *Af* biofilm.

## Materials and Methods

### Isolates

Any CF isolates from patient respiratory cultures were obtained after written informed consent, for biobanking of the patients’ specimens and subsequent use, approved by the Stanford Institutional Review Board. Other isolates were obtained following clinically indicated cultures. Twenty-six clinical isolates of *Pa* recovered from non-CF patients (n = 16 isolates), or CF patient sputum (n = 10), from Stanford University Hospital and clinics were evaluated. Among the CF isolates, five were mucoid colony phenotype variants [2,8,9] and five were non-mucoid colony phenotype variants. A list of all isolates studied, and their classification, is given in Tables 1 and 2. We were able to include a mucoid and a non-mucoid *Pa* isolate obtained from the same CF patient the same day, 2 non-mucoid *Pa* isolates from another CF patient 6 mos. apart, and 2 Pa isolates with different colonial morphologies from each of 2 non-CF patients obtained the same day, plus another *Pa* isolate from one of these patients one month later. *Af* isolate 10AF, a virulent non-CF patient isolate [49,50], was used as the reference *Af* isolate throughout this study. Nine sputum *Af* isolates, also identified by molecular methods to be *Af sensu stricto* [51], were obtained from non-CF patients in a previous study [51] and additionally studied.

**Table 1:** Clinical isolates.

Sample identification ID #	CIMR #	*P*. *aeruginosa* (Pa) phenotype	Clinical Laboratory ID # Valley Medical Center (VMC) or Stanford University (SU)	Specimen	Date of the positive Pa culture
1	14–75	Pa Non CF	VMC	Respiratory	1/28/2013
2	14–76	Pa Non CF	VMC	Respiratory	2/19/2013
3	14–77	Pa Non CF	VMC	Respiratory	2/19/2013
4	14–78	Pa Non CF	SU 60370871	Respiratory	2/17/2013
9	14–83	Pa Non CF	VMC	Respiratory	3/17/2013
12	14–86	Pa Non CF	SU 42266353	Respiratory	4/22/2013
13	14–87	Pa Non CF (Strain #1)	SU 21548242	Respiratory	8/10/2013
14	14–88	Pa Non CF (Strain #2)	SU 21548292	Respiratory	8/10/2013
16	14–90	Pa Non CF (Strain #1)	SU 27917939	Respiratory	9/24/2013
17	14–91	Pa Non CF (Strain #2)	SU 27917939	Respiratory	9/24/2013
19	14–93	Pa Non CF	SU 21548292	Respiratory	9/26/2013
20	14–94	Pa Non CF	SU 10790053	Non respiratory	8/14/2013
24	14–98	Pa Non CF	SU 41082579	Non Respiratory	10/02/2013
25	14–99	Pa Non CF (strain #2)	SU 22765499	Non respiratory	9/25/2013
26	14–100	Pa Non CF	SU 26308684	Respiratory	9/26/2013
27	14–101	Pa Non CF	SU 28674323	Non respiratory	9/23/2013
5	14–79	Pa CF Mucoid	SU 20060455	Respiratory	3/1/2013
11	14–85	Pa CF Mucoid	SU 60821238	Respiratory	2/21/2013
18	14–92	Pa CF Mucoid	SU 09710807	Respiratory	7/19/2013
21	14–95	Pa CF Mucoid	SU 7841943	Respiratory	9/17/2013
22	14–96	Pa CF Mucoid	SU 41053570	Respiratory	8/10/2013
7	14–81	Pa CF Non Muc	SU 09710807	Respiratory	1/19/2013
8	14–82	Pa CF Non Muc	SU 16242976	Respiratory	3/13/2013
10	14–84	Pa CF Non Muc	SU 7841943	Respiratory	2/01/2013
15	14–89	Pa CF Non Muc	SU 41053570	Respiratory	8/10/2013
23	14–97	Pa CF Non Muc	SU 16242976	Respiratory	9/6/2013

**Table 2:** *Aspergillus fumigatus* clinical isolates.

Sample identification ID #	CIMR #	*A*. *fumigatus* phenotype
1	13–55	CF
2	13–56	CF
3	13–57	CF
4	13–58	CF
5	13–59	CF
6	13–60	CF
7	13–61	CF
8	13–62	CF
9	13–63	CF
10	12–26	Non CF
11	12–30	Non CF
12	12–48	Non CF

*Af* conidia were obtained as follows: *Af* was taken from stock suspensions stored at -80°C and then grown for 4 days on Sabouraud Dextrose Agar (Becton Dickinson and Co., Sparks, MD) at 37°C. Conidia were harvested by gently washing with 0.05% Tween-80 (J.T. Baker Chemical Co., Phillipsburg, NJ) in 0.9% saline (Baxter Healthcare Corp., Deerfield, IL).

*Pa* stocks were maintained at -80°C in Microbank microbial storage vials (Pro-Lab Diagnostics, Richmond Hill, Ontario, Canada). Each frozen *Pa* stock culture was initially inoculated onto Trypticase Soy + 5% sheep blood agar plates (TSA; BBL, Becton Dickinson; subsequent studies indicated the absence of blood in the agar plates did not affect the activity of the *Pa* harvested) and incubated overnight at 37°C. *Pa* colonies were then picked, 1–2 loopfuls of the bacteria diluted in RPMI-1640 medium, and the suspension adjusted in the spectrophotometer at A610 to an absorbance of 0.35–0.40 with fresh RPMI-1640 medium. This corresponded to 10^9^/ml, with variation over a 3-fold range. A 1:30 dilution was then made in studies of direct *Pa* action on *Af* biofilm, or production of *Pa* culture supernatants, to be described. We found, in the studies to be described, that neither direct *Pa* action, nor *Pa* supernatant action, on *Af* biofilm was particularly sensitive to the size of the initial *Pa* inoculum in those studies, as variations of at least 10-fold in live *Pa* cells, and at least 33-fold in the inoculum used to make planktonic *Pa* supernatants, resulted in no significant differences in activity.

### Inhibition of planktonic *Af* growth

Inhibition of growth was assessed by using a conidial inoculum, following guidelines for susceptibility testing of filamentous organisms [52].

### *In vitro* model of biofilm development

Biofilms were formed by using a modified *in vitro* model described previously [53]. To form *Af* biofilm, sterile polystyrene disks (Biosurface Technologies, Bozeman, MT) were placed in 12-well tissue culture plates (Corning Inc., Corning, NY). Each well contained 3 ml of fresh RPMI-1640 medium (Lonza, Walkersville, MD) with 10^5^ *Af* conidia/ml.

**Fig 1:**
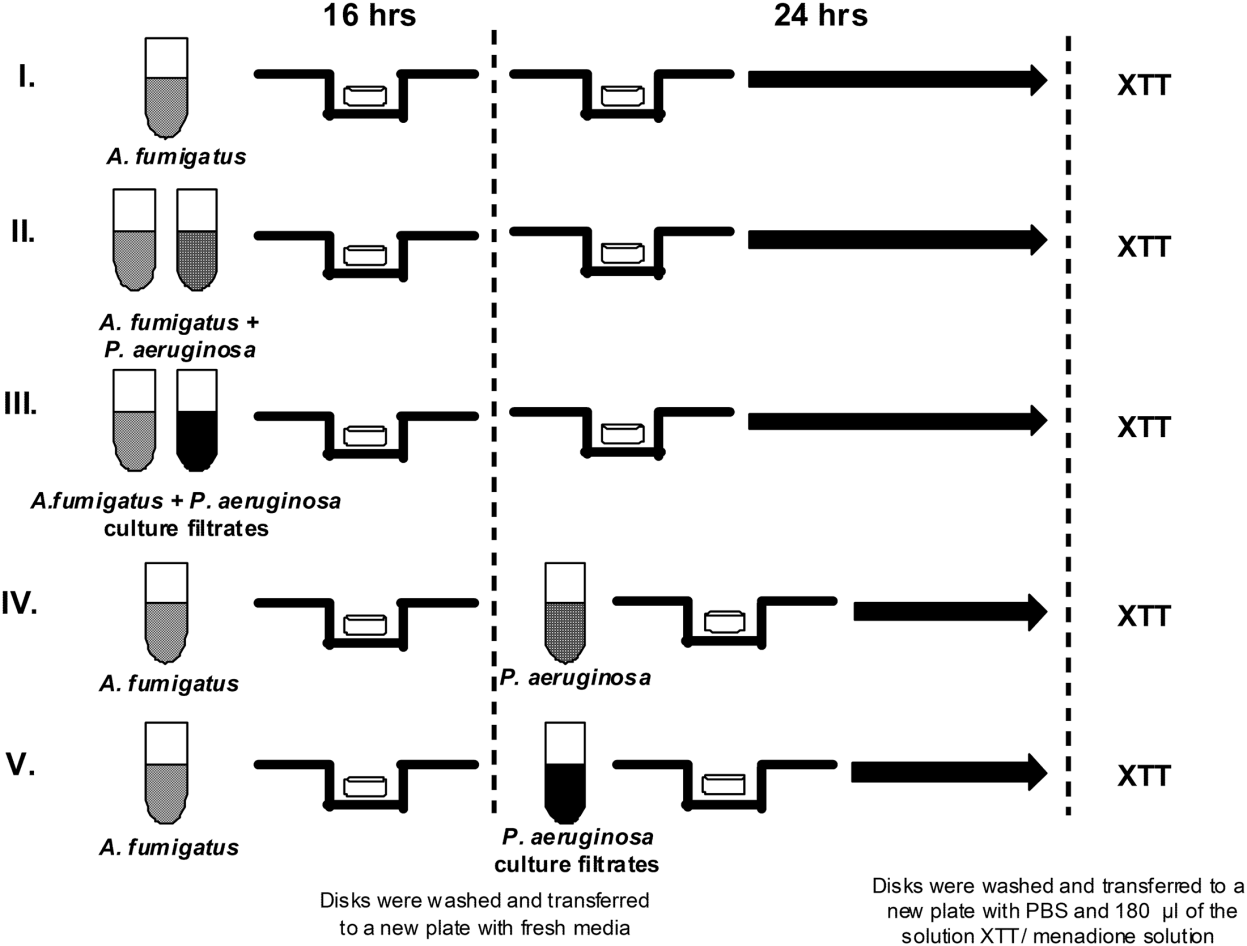
Experimental design (full details are provided in Methods). To form *Af* biofilm, polystyrene disks were placed in tissue culture plates with conidia and media. Disks were incubated to allow the conidia to attach. Following the attachment phase, disks were transferred to new plates containing and incubated for an additional 24 h (line I). Direct interaction of live *Pa* on *Af* biofilm formation: Suspensions of conidia and bacteria were combined in tissue culture plates for 16 h. Disks were then rinsed gently, transferred to new plates, and incubated for an additional 24 h (line II). Direct interaction of live *Pa* on preformed *Af* biofilm: Fungal biofilms were formed as described. After 16 h the disks were rinsed, transferred to new plates containing *Pa* suspension and incubated an additional 24 h (lines II and IV). *Pa* planktonic supernatant assay: To obtain planktonic culture filtrates, *Pa* suspension was incubated in conical tubes for 24 h. The spent medium was centrifuged to remove suspended cells or debris. The supernatant was filter sterilized and added to wells of a tissue culture plate previously inoculated with *Af* suspension. Fungal biofilms attached and formed. Disks containing biofilms were washed, transferred to a new plate, and incubated for an additional 24 h (line III). For the preformed biofilm assay, filtered supernatant was added to wells. Disks containing *Af* preformed biofilms were washed, transferred to the plate containing the bacterial filtrates, and incubated an additional 24 h. *Af* wells without bacterial supernatant were included as controls (line V). To obtain *Pa* biofilm filtrates, a suspension of *Pa* adhered to tissue culture flasks for 2 h (attachment phase). The flasks were rinsed, fresh RPMI-1640 added to the flask and adhered cells formed *Pa* biofilms for 22 h. The spent medium was then removed. *Af* conidia forming biofilms or preformed *Af* biofilms were challenged with the *Pa* biofilm culture filtrate (lines III and V).

Disks were incubated at 37°C for 16 h with shaking at 70 rpm, to allow the fungal cells to attach. Following the attachment phase, disks were gently rinsed in sterile saline (Baxter Healthcare Corp.), transferred to new plates containing fresh RPMI-1640 medium, and incubated for an additional 24 h at 37°C with shaking at 100 rpm (Fig 1I). Biofilm formation here, and in all experimental conditions to be described, was verified by inspection or with a light microscope [16].

### Direct interaction of live *Pa* on *Af* biofilm formation

Suspensions of fungal conidia (3 ml) and bacterial cells (0.1 ml), prepared as described above, were combined in 12-well tissue culture plates in fresh RPMI-1640 medium and incubated at 37°C for 16 h with shaking at 70 rpm. Thus, for the 3 x 10^5^ *Af* /well always involved in biofilm formation, the ratio of *Pa* to *Af* was 3.3 x 10^7^:3 x 10^5^/well. Disks were then rinsed gently with sterile saline, transferred to new plates containing fresh RPMI-1640 medium, and incubated for an additional 24 h at 37°C with shaking at 70 rpm (Fig 1II).

### Direct interaction of live *Pa* on preformed (i.e., established) *Af* biofilm

Fungal biofilms were formed as described above. After 16 h the disks containing the fungal preformed biofilms were rinsed gently in sterile saline, transferred to new plates containing *Pa* suspension (3 ml containing 3.3 x 10^7^ cells) prepared as described above, and incubated for an additional 24 h at 37°C with shaking at 70 rpm (Fig 1II and 1IV).

### *Pa* planktonic supernatant assay

A *Pa* suspension was prepared as described above in fresh RPMI-1640 medium. To obtain the planktonic culture filtrates, the bacterial suspension in fresh RPMI described above was incubated in 50 ml conical tubes (Falcon, Brookings, So. Dakota) for 24 h at 37°C with shaking at 70 rpm. The spent medium was removed, transferred to a new 50 ml conical tube, and centrifuged for 30 min at 2,000 x *g* to remove any suspended cells or debris. The planktonic supernatant was gently removed, filter sterilized (0.22 μm) (Fisherbrand, Pittsburgh, PA) and used. Filtered supernatant (1.5 ml) was added to selected wells of a 12-well tissue culture plate previously inoculated with 1.5 ml of the standardized *Af* suspension in fresh RPMI-1640. Fungal biofilms attached and formed as described above. Disks containing biofilms were washed 3 times with sterile saline and transferred to a new plate containing fresh RPMI-1640, and incubated for an additional 24 h at 37°C with shaking at 70 rpm (Fig 1III). Although “supernatants” and “filtrates” are used interchangeably in this paper, when any supernatants were tested they were always first filtered as described above; unfiltered supernatants were never used.

For the preformed biofilm assay, 1.5 ml of the filtered bacterial supernatant was added to selected wells of a 12-well tissue culture plate with 1.5 ml of fresh RPMI-1640. *Af* biofilms were prepared as described above. Disks containing these fungal preformed biofilms were washed 3 times with sterile saline, transferred to the plate containing the fresh media + the bacterial filtrates, and incubated for an additional 24 h at 37°C with shaking at 70 rpm. *Af* wells without bacterial supernatant were also included to serve as controls (Fig 1V).

To assay growth during *Pa* planktonic culture, the *Pa* planktonic suspension was prepared as described above, adjusted to 10^6^/ml, incubated as described above, and at the end of the 24 h, serial dilutions of the supernatant were inoculated onto TSA plates, incubated for 24 h at 37°C, and CFU enumerated.

### *Pa* biofilm filtrate assay

To obtain the *Pa* biofilm filtrates, a 25 ml suspension of *Pa* prepared as described above, adhered to 50 ml canted neck tissue culture flasks (BD Biosciences, San Diego) for 2 h at 37°C on a 100 rpm shaker incubator (attachment phase). The liquid was then removed and the flasks were rinsed gently 3 times with sterile saline. Twenty ml of fresh RPMI-1640 was added to the flask and adhered cells formed *Pa* biofilms for 22 h (total of 24 h), observed by the presence of a layer of bacterial growth on the inner surface of the flask. The spent medium was then removed and processed as described above. *Af* conidia forming biofilms or preformed *Af* biofilms were challenged with the *Pa* biofilm culture filtrate, as described above. Bacterial supernatant-free wells were also included as controls (Fig 1III and 1V). Either *Pa* planktonic or biofilm supernatants were used when prepared, although studies indicated no change in activity if stored refrigerated for at least one week.

### Studies with serum

For sets of experiments with *Af* and *Pa* cultures, to be described, fetal bovine serum (Gibco, Grand Island, NY) was added to the RPMI-1640 medium at each step to produce a 10% concentration.

### XTT assay

Either when conidia formed biofilm, or when preformed *Af* biofilm was studied, at the end of the challenge period with either live *Pa* cells or filtered *Pa* cultured supernatants (or control media), the *Af* biofilm was studied with the XTT assay. The tetrazolium salt, XTT (2,3-bis[2-methoxy-4-nitro-5-sulfophenyl]-2H-tetrazolium-5-carboxanilide inner salt) (Sigma, St. Louis, MO) was used to measure the metabolic activity of *Af*. XTT is reduced by a mitochondrial dehydrogenase to a water-soluble formazan product. *Af* biofilm discs were rinsed 3 times in sterile saline and transferred to fresh wells containing 3 ml sterile phosphate-buffered saline (PBS) (pH 7.3–7.5, Lonza). Menadione (Sigma) 0.85 gm was added to 5 ml acetone, and mixed in a 1:11 ratio of menadione to XTT (1 mg/ml). The XTT-menadione solution (180 μl) was added to each well and the plates were incubated in the dark for 2 h at 37°C. Following incubation, the contents of the wells were collected and centrifuged for 10 min at 13,300 x g. The pellet was then discarded and the absorbance at 490 nm of the supernatant was determined with a spectrophotometer (Genesys 20, Thermo Scientific, Waltham, MA). Although XTT is a measure of metabolic activity of cells, previous studies of *Af* have indicated XTT results are linear with mass, and equated XTT result with dry weight [54–56].

For the assay of live *Pa* cells co-incubated with *Af*, we determined that the XTT readings only reflected the *Af* by studying *Pa* similarly cultured without *Af*, showing there was no increase of XTT reduction compared to background without any microbes present.

### Confocal laser scanning microscopy (CLSM)

*Af* biofilms were formed on disks as described above. The biofilms were challenged with *Pa* supernatants obtained from a representative of each of the 3 *Pa* groups. After incubation at 37°C, the disks were washed three times in sterile PBS and stained using a fluorescent stain (FUN-1; Invitrogen Molecular Probes, Eugene, OR), prepared according to the manufacturer’s instructions. FUN-1 (1 μl) from a 10 mM stock was mixed in 1 ml of PBS. Staining was performed as previously described [11,13]. Three drops of the mixture were added on the top of the biofilm, which was then mounted on a glass slide and covered with a glass coverslip (22 × 22 mm). The disks were incubated for 45 min at 37°C in the dark. The FUN1 visualized the morphology of *Af* biofilm, a bright green cytoplasmic stain produced after passive diffusion. An additional analysis was made to visualize the effect of bacterial supernatant on the fungal biofilm metabolic activity. A red fluorescence, visualized from intravacuolar staining, was noted, which requires both plasma membrane integrity and metabolic capability (data not shown). Dead cells would have fluoresced bright yellow-green, with no red structures, and were not apparent in our images.

Sections on the xy plane were taken at 1 μm intervals along the z-axis to determine the depth of the biofilms. Microscopic visualization and image acquisition of biofilms were conducted at the Stanford Biofilm Research Center using an upright Leica TCDSP2 scanning confocal laser microscope (Leica Lasertechnik GmbH, Heidelberg, Germany) equipped with an argon/krypton laser and detectors, and filter sets for monitoring of green (excitation 480nm, emission 517nm) and red (excitation 633nm, emission 676). Images were obtained using a 63 x 1.4 Plan-APOChromat DIC (Leica, Heidelberg, Germany) objectives. Multichannel simulated fluorescence projection (SFP, a shadow projection) images and vertical cross sections through the biofilm were generated using the IMARIS software package (Bitplane AG, Zürich, Switzerland). Images were processed for display by using Photoshop software (Adobe, Mt. View, CA). Representative images were taken.

### Dose-response assay of filtrates

A dose response study was performed using one isolate from each of the 3 *Pa* groups. *Pa* planktonic and biofilm culture filtrates were obtained as described above. The culture filtrates or sterile distilled water were diluted in fresh RPMI supplemented with 10% FBS, so that the percent of fresh medium in each test situation was decreased as the spent medium or sterile distilled water increased. *Af* biofilms were formed in this mixed media, as described above, or planktonic *Af* growth was studied [52].

### Effect of temperature on filtrates

The *Pa* planktonic and biofilm culture filtrates were prepared as described above. In brief, the final cell-free supernatants were (a) heat treated at 56°C for 30 min and immediately used or (b) frozen at -80°C, stored for 6 days, and thawed. These filtrates were then tested compared to fresh filtrates against either *Af* conidia forming biofilm, or preformed biofilm, as described above.

### Effect of DNase I and Proteinase K on *Pa* supernatant activity

To determine whether *Pa* or phage DNA or a *Pa* protein(s) were involved in the inhibition of *Af*, we studied the effect of pretreatment of *Pa* culture filtrates with DNase I (Life Technologies, Grand Island, NY) or Proteinase K (Sigma) and measured the filtrate inhibitory activity on *Af* biofilm formation (Fig 1I). *Pa* biofilm culture filtrates were prepared as described using a CF non-mucoid *Pa* isolate, *Af* biofilm metabolic activity was measured using the XTT assay.

*Pa* culture filtrates were treated with DNase I or Proteinase K for 2 hours at 37°C. Ethylenediaminetetraacetic acid disodium salt dehydrate (EDTA) (Sigma) was used to inactivate the DNase I activity. Experiments with DNase I treatment involved growing the *Af* for 16 hours in the presence of (a) *Pa* filtrate, (b) DNase I (final concentration 2 U/ml) + *Pa* filtrate, (c) DNase I + EDTA (final concentration 20mM) + *Pa* filtrate, (d) EDTA + *Pa* filtrate, or (e) EDTA alone or (f) DNase + EDTA (in fresh RPMI medium). These were followed by 24 h growth in fresh RPMI-1640 alone.

For Proteinase K pretreatment, conidia were incubated with (a) *Pa* filtrate, (b) Proteinase K (final concentration 50 μg/ml) + *Pa* filtrate, or (c) medium treated with Proteinase K.

### Materials for other characterization studies

A 30,000 MW cutoff filter was obtained from Millipore Ltd., Tullagreen, Ireland.

Elastolytic production of *Pa* isolates was assessed as previously described [57].

FeCl_3_ was obtained from Sigma.

### Statistical analyses

**Fig 2:**
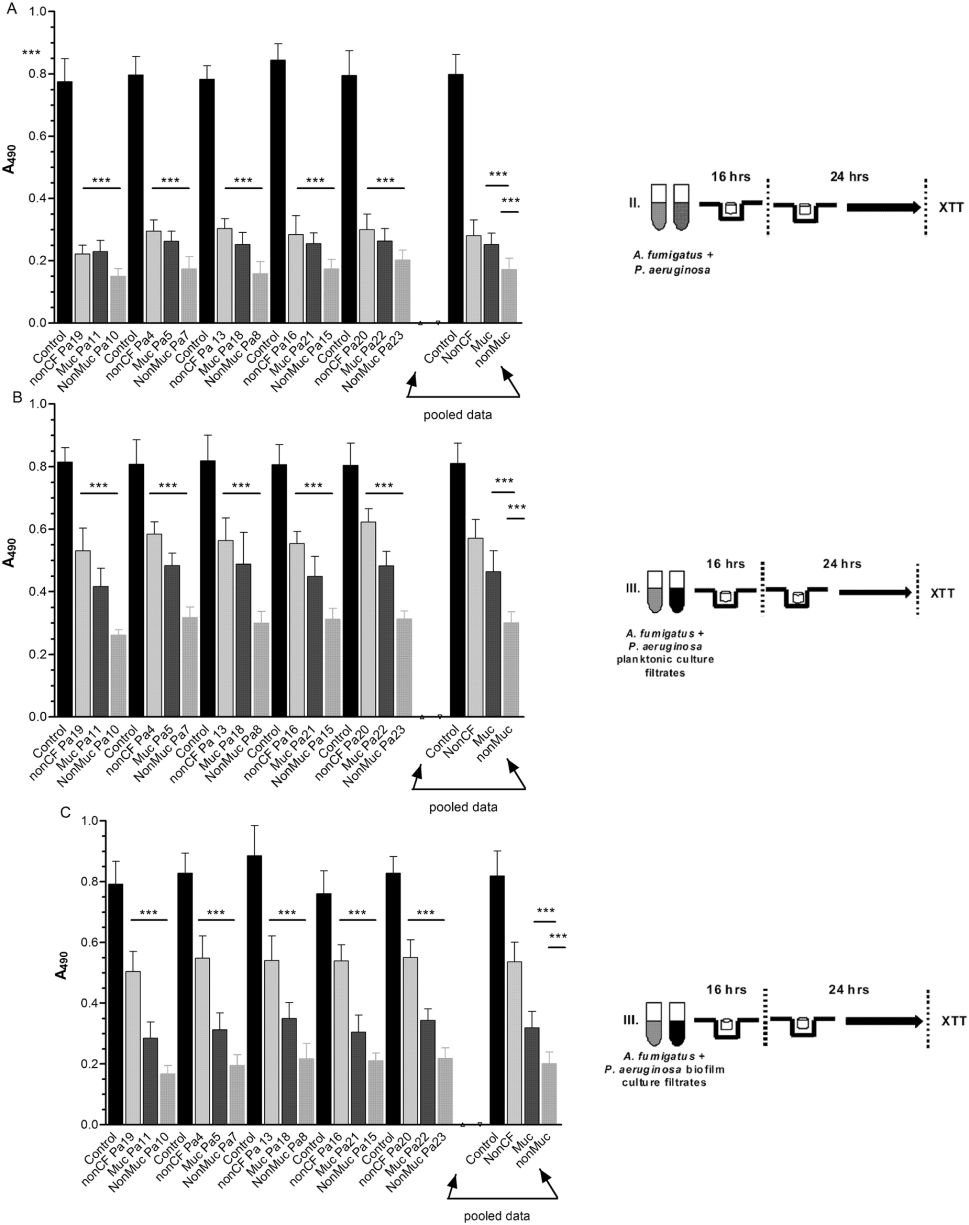
*Pa* inhibition of *Af* during biofilm formation. The data presented show the activity of each live *Pa* isolate or planktonic- or biofilm-generated *Pa* culture filtrate on *Af* during formation of biofilm by *Af*. Individual isolate results are shown in comparison with their respective *Af* controls (n = 6 for each). There were five non-CF, five mucoid CF and five non-mucoid CF isolates tested; isolate designations are shown on the x-axis. The bars on the far right of each panel represent the pooled data for each type of isolate (n = 30). Bars represent the mean ± SD of the XTT reduction read at 490 nm. (A) *Af* conidia were exposed to live *Pa* cells for 16 h. (B) *Af* conidia were exposed to *Pa* planktonic supernatant for 16 h. (C) *Af* conidia were exposed to *Pa* biofilm supernatant for 16 h. The resulting readings were determined. “XTT metabolic activity” refers to the spectrophotometric absorbance of the formazan reduction product of XTT at 490 nm. Assays were performed in blocks with two runs of triplicates done on different days, for each *Pa-Af* combination. Panel (A) All individual *Pa* isolates, noted on the x-axis, were significantly inhibitory compared to controls (*P*<0.001). The four bars on the right side show the pooled data. CF mucoid and CF non-mucoid cells were more inhibitory than non-CF *Pa* cells (*P*<0.001, both comparisons), with CF non-mucoid cells more inhibitory than CF mucoid cells (*P*<0.001). Panel (B) Planktonic culture filtrate from all individual *Pa* isolates, were significantly inhibitory compared to controls (*P*<0.001). Pooled data analysis showed both CF mucoid or CF non-mucoid isolate culture filtrate was more inhibitory than that from non-CF isolates (*P*<0.001, both comparisons) and non-mucoid was more inhibitory than mucoid (*P* < 0.001). Panel (C) Culture filtrates from all *Pa* isolates grown as biofilm were significantly inhibitory (*P*<0.001). Pooled data showed culture filtrate from CF non-mucoid isolates under biofilm conditions was more inhibitory than that from CF mucoid or non-CF isolates under the same conditions (*P*<0.001, both comparisons), and that non-mucoid CF filtrates were more inhibitory than mucoid isolate filtrates (*P* < 0.001).

**Fig 3:**
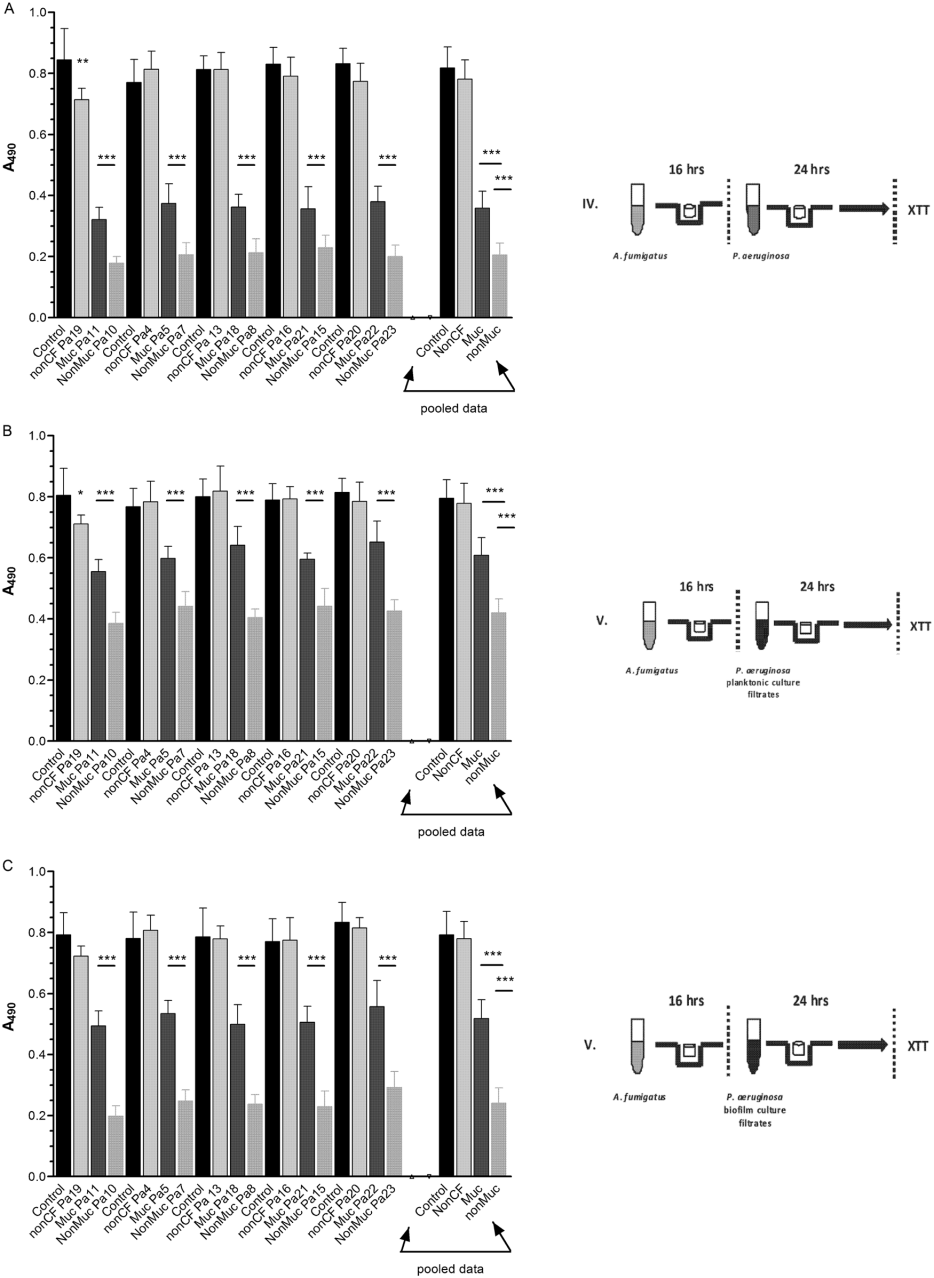
*Pa* inhibition of *Af* biofilm. The data presented show the activity of each live *Pa* isolate or planktonic or biofilm generated culture filtrate on preformed biofilm of *Af*. Individual isolate results are shown in comparison with their respective *Af* controls (n = 6 for each). There were five non-CF, five mucoid CF and five non-mucoid CF isolates tested; isolate designations are shown on the x-axis. The bars on the far right of each panel represent the pooled data for each isolate (n = 30). Bars represent the mean ± SD of the XTT reduction at read at 490 nm. Panel (A) *Af* preformed biofilms exposed to live *Pa* cells for 24 h. (B) *Af* preformed biofilms were exposed to *Pa* planktonic spent supernatant for 24 h. (C) *Af* preformed biofilms were exposed to *Pa* biofilm spent supernatant for 24 h. Each data point represents the XTT metabolic activity obtained spectrophotometrically at 490 nm. Assays were performed in triplicate and the results are pooled from two experiments for each *Pa-Af* combination. The asterisk indicates a significant *P* value (< 0.001) for the XTT metabolic activity compared to the positive control, using the same analytic methodology as in Fig 2. Panel (A) Individual *Pa* isolate comparisons showed that only a one non-CF isolate, Pa19, inhibited (*P*<0.01), whereas all mucoid or non-mucoid CF isolates were inhibitory (*P*<0.001, all comparisons). Pooled data analysis showed that both mucoid and non-mucoid CF isolates were significantly inhibitory (*P*<0.001, both comparisons) and that non-mucoid CF isolates were more inhibitory than non-CF or mucoid *Pa* isolates (*P*< 0.001, both comparisons). Panel (B) Planktonic spent medium from a single non-CF isolate, Pa19, was inhibitory (*P*<0.05), whereas all mucoid and non-mucoid CF were significantly inhibitory (*P* < 0.001). Pooled data analysis showed that planktonic culture filtrate from CF non-mucoid or mucoid isolates was inhibitory (*P*<0.001, both comparisons). Non-mucoid CF isolates were more inhibitory than mucoid CF isolates (*P*<0.001). Panel (C) Biofilm culture filtrate from non-CF isolates of *Pa* was not inhibitory, whereas the culture filtrate from each mucoid or non-mucoid CF *Pa* isolates was inhibitory (*P*< 0.001, all comparisons). Pooled data analysis showed that biofilm culture filtrate from CF non-mucoid or mucoid isolates was inhibitory (*P*<0.001). Non-mucoid CF isolate filtrates were more inhibitory than mucoid CF isolates (*P*<0.001).

Where *n* is not stated, each experiment was performed at least two times, with triplicate wells each time. Data from experiments using XTT as the assay parameter were collected in blocks. Each block was comprised of n = 6 values derived from of two sets of triplicate values collected on different days. For the data presented in Figs 2 and 3 statistical analyses were performed as follows. Each condition had five blocks of data, which were comprised of controls, nonCF *Pa*, mucoid *Pa* and nonmucoid *Pa* (*Pa* was either live cells or spent culture filtrates as indicated). Each block of four conditions was analyzed separately using one-way analysis of variance (ANOVA) followed by a Tukey’s post-hoc test to adjust for the multiple comparisons being performed. Prior to pooling, the data in all blocks were verified to be normally distributed and to have homogenous variances. An initial ANOVA comparison of the five blocks for each condition (e.g., controls) showed the blocks for that condition (e.g., controls) not be significantly different. These results allowed us to pool the data for each condition with a result of n = 30 each. The pooled data were analyzed using one-way ANOVA with a Tukey’s post-test. For comparisons across live cell versus planktonic or biolfilm supernatants, control values were first compared and determined to be no different, which was followed by ANOVA and Tukey’s comparisons of the experimental sets. All statistical analyses were done using GraphPad Prism (GraphPad Software, Inc. La Jolla, CA). Statistical significance was considered *P*<0.05.

## Results

A summary of all the studies to now be described is in S1 Table. Our primary aim in these studies was that of a population survey to determine whether *Pa* from CF or non-CF patients showed differences in their interactions with *Af*. Thus we designed studies to assess *Pa* from non-CF patients compared with mucoid and nonmucoid *Pa* from CF patients. Two experimental designs were used. The first was that of the effects of *Pa* on the development of biofilm by *Af* conidia and the second was that of the effects of *Pa* on preformed *Af* biofilm. In all of these studies we assessed the effects of live *Pa* cells and the effects of spent culture filtrates from *Pa* planktonic or biofilm growth and initially relied on the parameter of metabolic activity of Af, based on XTT reduction. For studies in Figs 2 and 3, five non-CF isolates were selected randomly from the 16 available, and compared to the CF mucoid and non-mucoid isolates.

### Inhibition by *Pa* cells or *Pa* culture filtrates during *Af* biofilm formation

The experimental design is shown in Figs 1 and 2, exposing the *Af* conidia to live Pa cells or filtrates for 16 h followed by an additional 24 h of biofilm growth before metabolic assay of XTT reduction.

Fig 2A summarizes the activity of the *Pa* cells during *Af* biofilm formation, as assessed by metabolic activity. Comparisons of effect of individual isolates of *Pa* on *Af* with their respective *Af* control showed that each isolate significantly inhibited Af during the formation of Af biofilm (*P*<0.001 all comparisons). Pooling of the 5 blocks of data showed that all three groups of *Pa* isolates significantly inhibited fungal biofilm formation (each group, *P*<0.001). Of interest, these data indicate that the inhibitory effect of CF mucoid *Pa* or CF non-mucoid isolates was higher compared to the effect of the non-CF isolates (*P*<0.001, both comparisons), and CF non-mucoid cells were more inhibitory than CF mucoid cells (*P*<0.001).

Fig 2B shows the *in vitro* activity of culture filtrates from *Pa* grown under planktonic conditions on the fungal biofilm formation, as assessed by metabolic activity. Comparisons showed that the planktonic culture filtrates of each individual isolate of *Pa* significantly inhibited *Af* during biofilm formation in comparison with controls (*P*<0.001). Similarly, the pooled data showed that the planktonic culture filtrates of each of the three groups of *Pa* isolates significantly inhibited *Af* (each, *P*<0.001). The inhibitory effect on *Af* biofilm formation by the culture filtrates from CF mucoid or CF non-mucoid *Pa* isolates was higher compared to the effect of the non-CF filtrates (*P*<0.001, both comparisons), and culture filtrates from CF non-mucoid *Pa* cells were more inhibitory than those from CF mucoid cells (*P*< 0.001).

The results of incubation of the conidia with culture filtrates obtained from *Pa* isolates grown under biofilm conditions are shown in Fig 2C. The culture filtrates of all isolates significantly inhibited Af attempting to form biofilm as compared to their respective controls (*P*<0.001, all comparisons). Examination of the pooled dated showed that biofim culture filtrates of *Pa* isolates from CF or non-CF patients significantly inhibited the metabolic activity of *Af* in biofilm development compared to the controls (all three groups, *P*<0.001). In addition, culture filtrates from CF mucoid or non-mucoid *Pa* isolates were significantly more inhibitory than those from non-CF isolates (*P*<0.001, both comparisons); CF non-mucoid *Pa* isolates biofilm culture filtrates were more inhibitory than those from mucoid isolates (*P*<0.001).

Whereas biofilm formation appears inhibited in the preceding studies, and in CLSM studies and studies of preformed *Af* biofilm to be described, we cannot completely rule out that differences in *Af* biofilm *formation* are a result of growth inhibition of the fungus. Studies (described below) of *Pa* inhibition of, by contrast, purely planktonic growth of Af, where there were not significant differences between the three *Pa* groupings, suggest the inhibitory differences of *Pa* groups relates more to the effect on *Af* biofilm formation.

### Effect of live *Pa* cells or culture filtrates on preformed *Af* biofilm

The experimental design for these studies is shown in Figs 1 and 3, that of allowing *Af* to form biofilm for 16 h and then exposing this biofilm to live *Pa* or culture filtrates for an additional 24 h before XTT assessment of *Af* metabolic activity. Assessment of the inhibitory effects of the live *Pa* cells showed that only a single non-CF isolate, *Pa*19, significantly inhibited the preformed *Af* biofilm (*P*<0.01), whereas the other four non-CF isolates had no significant effect (*P*>0.05). Compared to preformed *Af* biofilm controls, all isolates of non-mucoid or mucoid live *Pa* from CF patients significantly inhibited the metabolic activity of preformed *Af* biofilm (*P*<0.001). Pooling of the five blocks of data showed that non-CF isolates were not inhibitory, whereas mucoid *Pa* isolates and non-mucoid CF isolates significantly inhibited the metabolic activity of the preformed *Af* biofilm in comparisons to controls or non-CF isolates of *Pa* (*P*<0.001, both comparisons). Non-mucoid CF isolates were more inhibitory than mucoid CF isolates (P<0.001) (Fig 3A).

Fig 3B shows the activity of planktonic *Pa* culture filtrates on preformed *Af* biofilm. Comparisons of individual culture filtrate activity from individual isolates showed that a single non-CF isolate, *Pa*19, filtrate was inhibitory (*P*<0.05), whereas all those from individual filtrates from non-mucoid or mucoid *Pa* from CF patients, grown under planktonic conditions, significantly inhibited the oxidative metabolism of preformed *Af* biofilm (*P*<0.001, all comparisons). The results of analysis of the pooled data showed non-CF planktonic culture filtrates had no inhibitory activity, whereas planktonic filtrates from mucoid or non-mucoid *Pa* isolates were more inhibitory than controls or filtrates from non-CF isolates (*P*< 0.001, both comparisons). Non-mucoid CF isolate filtrates were more inhibitory than mucoid CF filtrates (P<0.001).

Fig 3C shows the activity that filtrates from cultures of biofilm-grown *Pa* isolates had on preformed *Af* biofilm. No inhibitory activity was exhibited by the non-CF biofilm filtrates (*P*>0.05), whereas all biofilm filtrates from mucoid or non-mucoid CF *Pa* from CF patients significantly inhibited preformed *Af* biofilm, compared to preformed *Af* biofilm controls (*P*<0.001). Similarly, the comparisons of the pooled data showed that non-CF isolate biofilm filtrates were not inhibitory and both mucoid and non-mucoid isolate biofilm filtrates were more inhibitory than controls or filtrates of non-CF isolates (*P*<0.001, both comparisons). Again, non-mucoid CF isolate biofilm filtrates were more inhibitory than those of mucoid CF isolates (P<0.001).

### Effect of *Pa* growth on inhibition by *Pa* supernatants

We felt that the effects of the Pa culture filtrates might plausibly be due to differences in growth of the Pa isolates, that isolates with better growth could result in more inhibitory compounds secreted to the culture filtrates. For these and subsequent studies, we chose one representative isolate from each *Pa* group (non-CF (*Pa*19), non-mucoid CF (*Pa*10) and mucoid CF (*Pa*11))(Figs 2 and 3). Each was grown planktonically and the 24 h growth quantitated as described (Methods). A 10^6^ inoculum of each grew to 3.2 x 10^8^ in the case of the two CF isolates, and 3.3 x 10^8^ for the non-CF isolate. Thus differences in the inhibitory power of the supernatants appear not explained by differences in growth among these isolates.

### Minimal effect of serum on inhibition

Because components of serum can also be present where there is local inflammation, such as in the airways, to more closely mimic in vivo conditions it is of interest to know whether the interactions described above might be different in the presence of serum. For these studies, serum was added to the medium in all steps (Fig 1), as indicated in Methods, and all 16 non-CF *Pa* isolates were used, as well as the mucoid and non-mucoid CF isolates.

Overall, the results were virtually the same as those from the studies done in the absence of serum. However, there were exceptions. The *Pa* live mucoid cells, or the filtrates of such cultures grown planktonically or as *Pa* biofilm, were, with serum present, not statistically significantly inhibitory, as assessed metabolically, to *preformed Af* biofilm. Specifically, absent that one exception, the significance and conclusions with (a) mucoid *Pa* and *Af* biofilm *formation*, or (b) non-CF *Pa* or non-mucoid CF *Pa*, as live cells or culture supernatants (the supernatants from cultures grown planktonically or as *Pa* biofilm), were the same as the results in the absence of serum, when tested against either (c) *Af* biofilm formation or (d) preformed *Af* biofilm, Again, non-mucoid *Pa* isolates were most inhibitory in all conditions, and preformed *Af* biofilm the most resistant. None of the 16 non-CF *Pa* isolates behaved differently than the rest of that group (data not shown).

In additional experiments (using only the 3 representative *Pa* isolates, as described in the preceding section, from each *Pa* group), we omitted serum from *Pa* while generating supernatants, but added serum during Af biofilm formation or preformed *Af* biofilm development. We found the same results, as described, as when serum was present or absent in *all* phases.

Overall, we conclude that serum is not an important enhancing or decremental factor in *Pa* generation of inhibition in supernatants, nor a factor in the effect on *Af*.

### Comparison of *Pa* filtrates grown planktonically or as biofilm

All of the studies done with or without serum appeared to indicate that there were differences in inhibitory activity between Pa planktonic and biofilm supernatants. Because there were few experiments, at this point, where these two types of *Pa* filtrates were compared directly to each other, we approached the comparison, for the studies in Figs 2 and 3, in the statistical fashion described in the Methods section. For the non-CF and the mucoid CF *Pa*’s, planktonic and biofilm supernatants inhibited *Af* biofilm formation. However, there was no difference between the planktonic and biofilm culture filtrates from the non-CF *Pa*, whereas *Pa* biofilm supernatants from mucoid and non-mucoid CF *Pa* were more inhibitory than planktonic supernatants (*P*<0.001, both comparisons). The supernatants of non-mucoid CF *Pa* were inhibitory to both *Af* biofilm formation and preformed *Af* biofilm, and *Pa* biofilm supernatants were more inhibitory than planktonic supernatants in both cases (*P*<0.001 for both comparisons). For comparison, a second analysis examined only the studies done in the presence of serum, and with all 16 non-CF isolates. For this second analysis we first compared the controls from each experiment where *Pa* isolates had been studied (in which only one type of *Pa* supernatant was used per experiment) by one-way ANOVA to assure the *Af* control results were not different in those experiments. These analyses showed that there were no significant differences in the *Af* controls. With this assurance we then compared the inhibition results of the three types of *Pa* for the two types of *Pa* supernatants. This allowed 20 experiments with the non-CF *Pa*’s and 10 experiments each with mucoid and non-mucoid CF *Pa* to be studied. The conclusions were the same as the prior statistical analysis, that of the studies done in the absence of serum.

These statistical differences in planktonic and biofilm *Pa* filtrates can also be suggested by visual comparison of Figs 2 and 3, although these differences were more marked when serum was present. In some subsequent experiments, under special conditions to be described subsequently (e.g., comparisons with *E*. *coli*), there *were* direct comparisons of the two kinds of *Pa* supernatants, and supernatants from planktonically grown *Pa* were never more inhibitory than supernatants from biofilm-grown *Pa*, regardless of the source of the *Pa*.

### Confocal microscopy analysis

All the preceding studies assessed metabolic effects on biofilm. We now assessed effects on the physical biofilm itself.

### Effects on biofilm thickness

**Fig 4:**
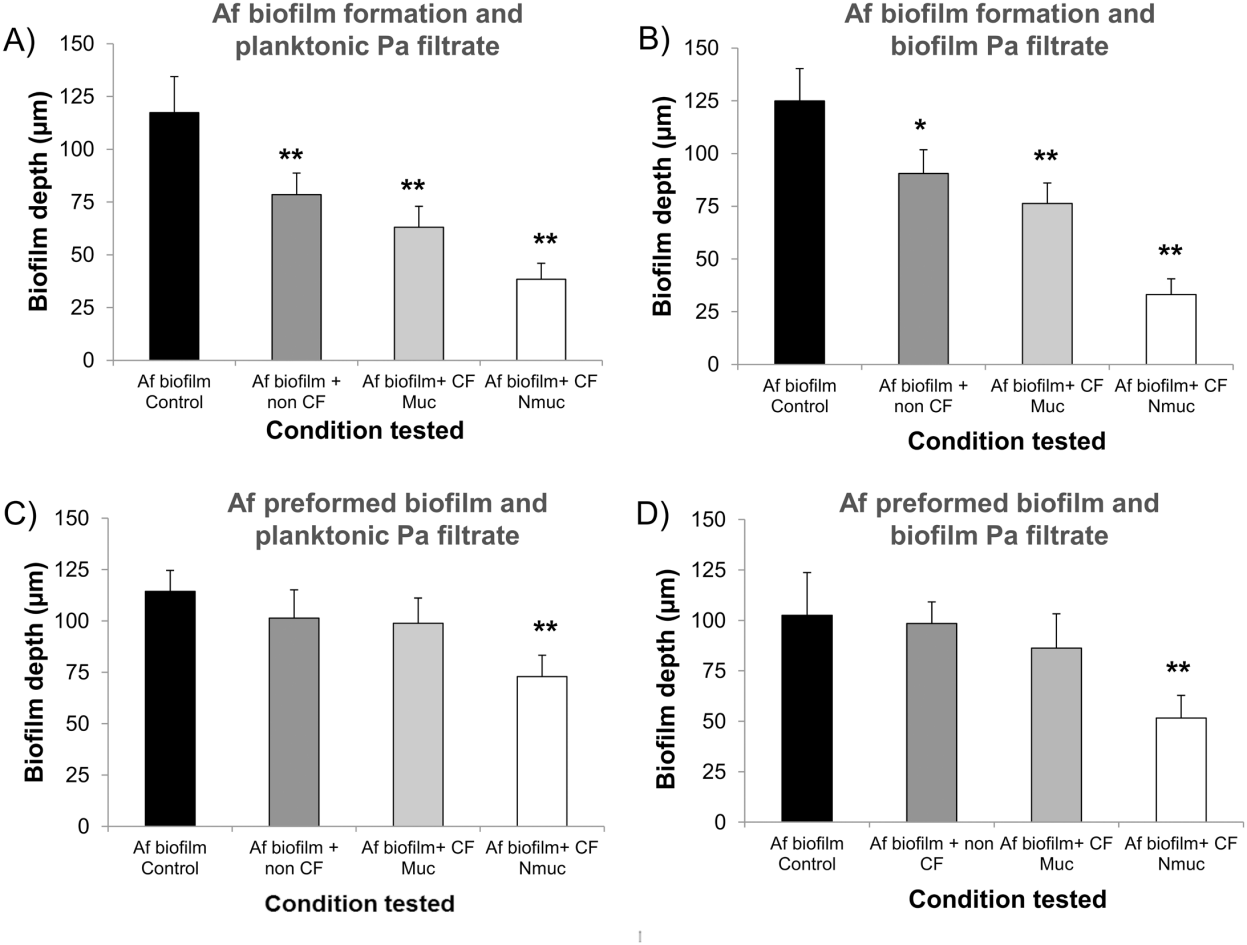
*Pa* supernatant affects *Af* biofilm thickness. *Af* biofilms were formed and stained as described. Sections of the xy plane were taken at 1 μm intervals along the z-axis to determine the depth of the biofilms. (A) *Af* conidia were exposed to planktonic *Pa* spent supernatant or (B) biofilm *Pa* spent supernatant, for 16 h. (C) *Af* preformed biofilms were exposed to planktonic *Pa* spent supernatant or (D) biofilm *Pa* spent supernatant, for 24 h. Assays were performed in triplicate and images were taken from three different fields from each sample. The results are representatives of two different experiments for each *Pa-Af* combination. One asterisk indicates a *P* value (<0.01), and two asterisks indicates a *P* value (<0.001) for the biofilm thickness compared to the untreated control.

The effect of the *Pa* spent culture filtrates on *Af* biofilm thickness and morphology were assessed using CLSM. The thickness results for the *Af* biofilm after conidia exposure for 16h to the *Pa* spent supernatants from the representatives of all three bacterial phenotypes are shown (Fig 4). The spent supernatant obtained from all three *Pa* phenotypes, after *Pa* growth planktonically or as biofilm, resulted in a significant reduction of the fungal biofilm thickness compared to the untreated control (Fig 4A and 4B). Treatment with the spent supernatants from the non-CF and the CF mucoid isolates had no significant effect on the thickness measurements of preformed *Af* biofilm, whereas the supernatant of the non-mucoid *Pa*, grown either planktonically or as biofilm, was inhibitory (Fig 4C and 4D).

### Effects on Af biofilm formation

**Fig 5:**
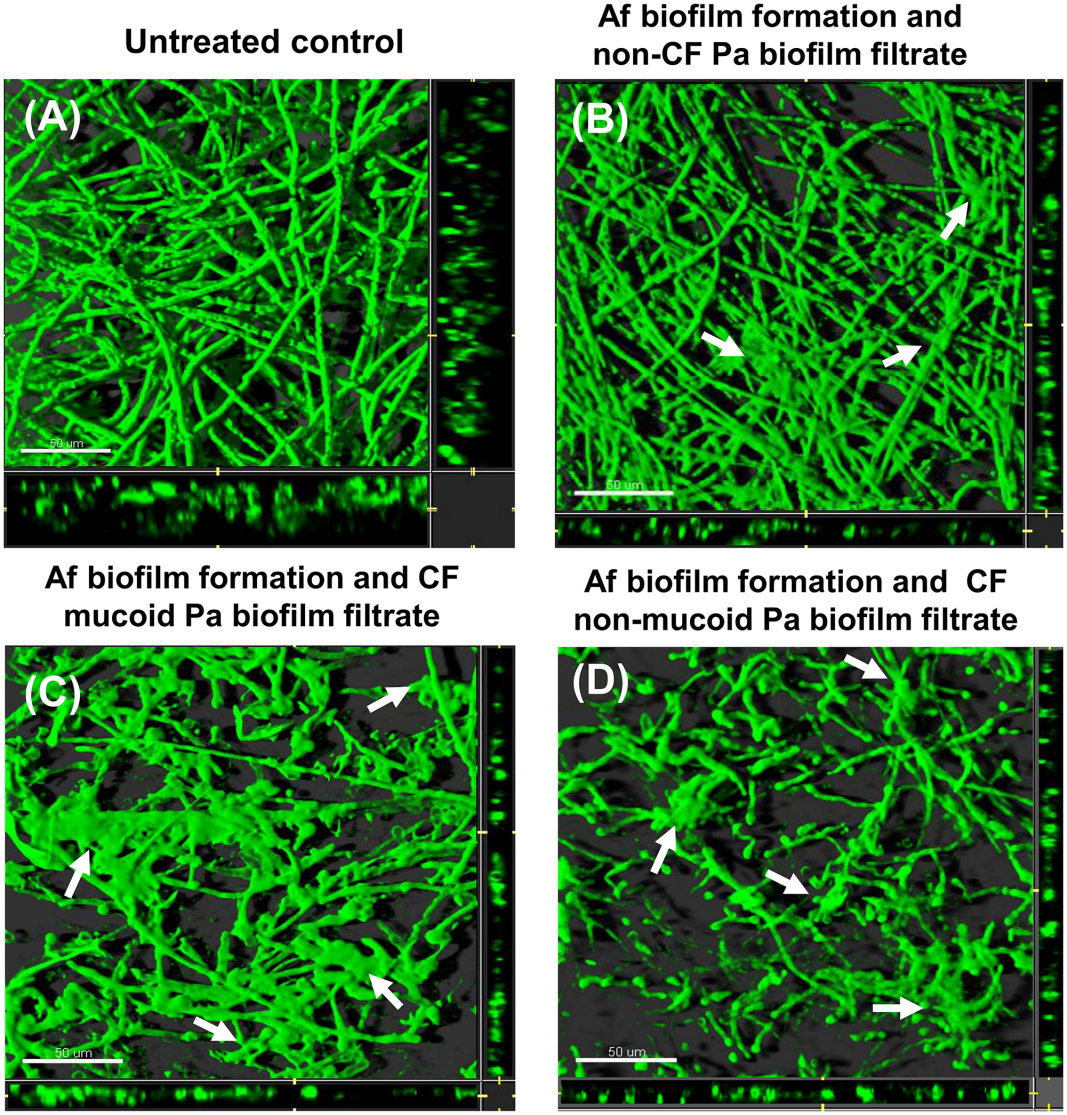
CLSM images of *Af* biofilm after challenging conidia for 16h with biofilm *Pa* spent supernatant. Horizontal (*xy*) view of reconstructed 3-dimensional images of FUN1-stained biofilms, with filter set to capture green fluorescence. Thickness of the biofilm can be observed in the side view of the reconstruction (extreme right and lower panels in each picture). (A) Untreated control. (B) *Af* conidia exposed to spent supernatant of a non-CF *Pa* grown as biofilm for 16 h, or (C) exposed to mucoid CF *Pa* biofilm spent supernatant or (D) exposed to non-mucoid CF *Pa* biofilm spent supernatant. Arrows show “bulge-like” structures and deposition of amorphous material in treated cultures. Magnification, ×63. Bar, 50 μm.

**Fig 6:**
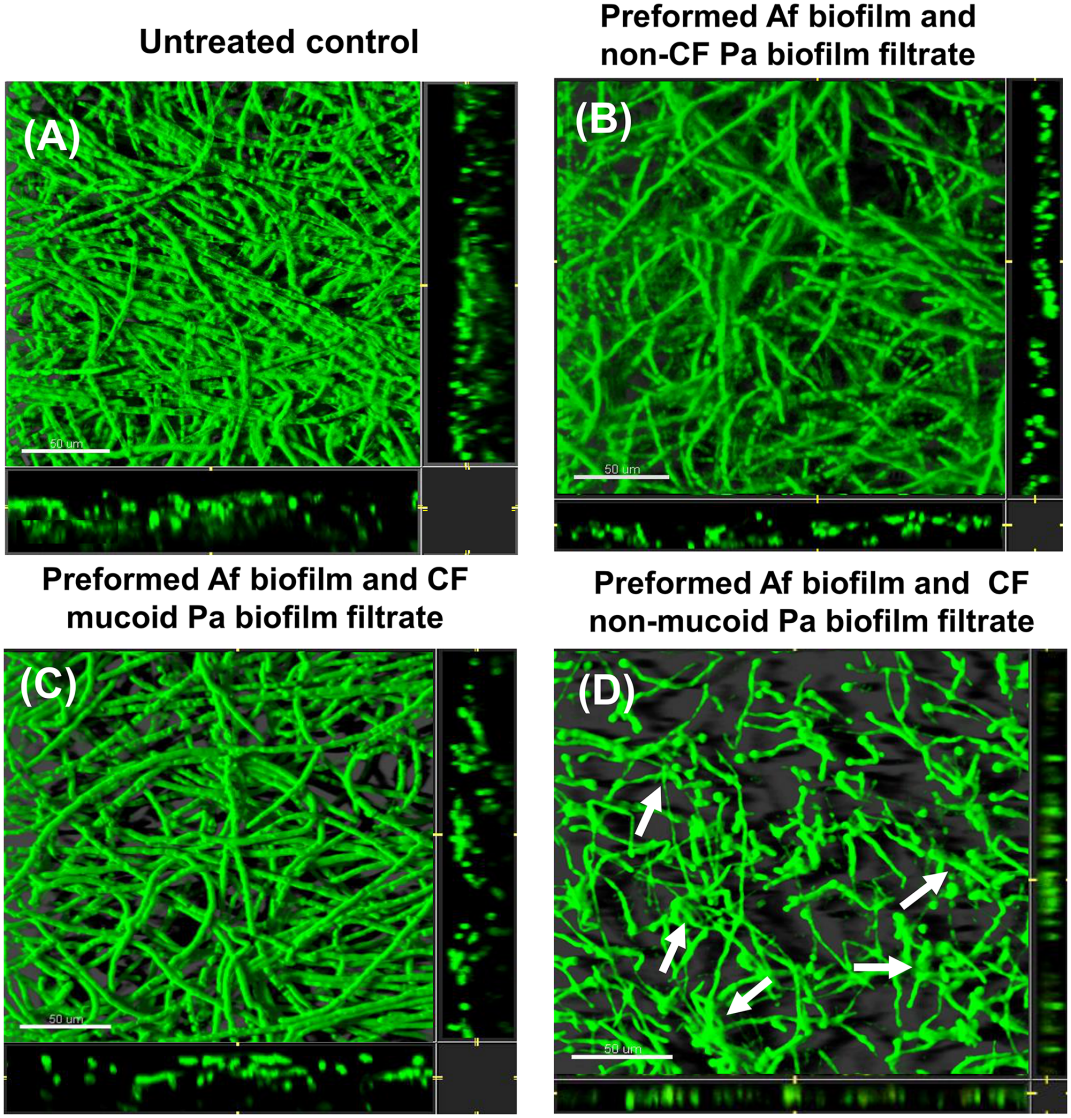
CLSM images of *Af* biofilm after challenging preformed *Af* biofilm for 24 h with *Pa* biofilm spent supernatant. Horizontal (*xy*) view of reconstructed 3-dimensional images of FUN1-stained biofilms, with filter set to capture green fluorescence. Thickness of the biofilm can be observed in the side view of the reconstruction (extreme right and lower panels in each picture). (A) Untreated control. (B) *Af* preformed biofilm exposed to spent supernatant of a non-CF *Pa* biofilm spent supernatant for 16 h or (C) exposed to mucoid CF *Pa* biofilm spent supernatant or (D) exposed to non-mucoid CF *Pa* biofilm spent supernatant. Arrows show the amorphous structures distributed throughout the intertwined altered filamentous networks in treated cultures. Magnification, ×63. Bar, 50 μm.

Untreated biofilms showed an architecture formed by a dense filamentous multicellular structure with acute-angle dichotomous branching (Figs 5A and 6A). The *Pa* supernatants’ effect on *Af* biofilm was also observed in the morphology studies using CLSM. Fig 5B, 5C and 5D show the effect of *Pa* culture filtrates on *Af* biofilm formation. Non-CF *Pa* spent supernatant, from *Pa* grown as biofilm, resulted in reduction in filamentation and presence of some “glued” hyphae, without a clear separation of the filamentous elements. A similar morphology pattern was observed when the conidia were treated with planktonic culture filtrates from the same strain (data not shown). Treatment with culture filtrates from the CF mucoid strain culture, grown under biofilm condition, resulted in decreased number of hyphae and presence of some "bulging" structures (possibly conidial remnants) distributed throughout the disperse filaments, and “glued” hyphae (Fig 5C). The CF non-mucoid *Pa* biofilm filtrate resulted in severe effects on hyphal structure, with loss of filamentation, and hyphal tips or branch points appeared to be thin. Several bulges and deposition of amorphous material were also observed. Treatment with planktonic spent supernatant from the CF non-mucoid isolate was associated with a disruption of the hyphae structure and presence of surface collapse and glued filaments (data not shown).

### Effects on preformed biofilm

As was suggested by the biofilm thickness data, treatment of *preformed Af* biofilm with the spent supernatants from the non-CF and the CF mucoid isolates were not associated with important morphology changes (Fig 6B and 6C). There was a prominent morphology change observed comparing the untreated *Af* control and treatment with the CF non-mucoid *Pa* strain grown under biofilm conditions (Fig 6D). We observed severe effects on hyphal structure, with loss of filamentation, and hyphal tips and branch points appeared to be thin after the treatment. Moreover, we observed amorphous structures distributed throughout the intertwined defective filamentous networks.

### MW characterization of the inhibitors in filtrates

To better understand the nature of the inhibitor(s) present in the *Pa* filtrates, we performed a series of studies to assess their physical and chemical nature.

To determine whether the inhibitors in filtrates were related to small molecular species, a sample of inhibitory filtrate from the non-mucoid *Pa* biofilm culture was passed through an ultrafilter that now excluded all materials >30,000 MW, and tested against *Af* conidia forming biofilm, in comparison to the same filtrate not further manipulated (as in Fig 1III). These two samples inhibited *Af* biofilm formation (*P*<0.001), and were not different from each other. We conclude that the inhibitory component(s) in *Pa* filtrates appear to be <30,000 MW species.

### Specificity

As a brief inquiry into the specificity of the culture filtrates of *Pa* inhibiting *Af*, we grew *E*. *coli* ATCC 43888 under identical conditions as we had the *Pa* isolates, and collected these filtrates in the same manner. Supernatant from the non-mucoid *Pa* was included in these experiments as a positive control. The filtrates of the *E*. *coli*, either grown planktonically or as biofilm, did not significantly inhibit *Af* conidia forming biofilm (conditions under which, in contrast, all *Pa* filtrates tested could inhibit; see Fig 2), and the positive *Pa* control filtrate did significantly inhibit. The filtrates of the *E*. *coli*, either grown planktonically or as biofilm, also did not inhibit preformed *Af* biofilm (data not shown).

To ascertain whether the reference *Af* isolate, 10AF, was representative, three and nine other non-CF *Af* isolates were tested, by challenging formation of *Af* biofilm from their conidia, or preformed Af biofilm, respectively (Fig 1II and 1IV, respectively), by live *Pa* cells of the non-mucoid CF *Pa*. Isolate 10AF was assayed concurrently. The XTT activity of all untreated *Af* biofilms formed were not significantly different, and all were markedly inhibited (*P*<0.001) by the addition of the *Pa* cells (data not shown).

We conclude that not all bacteria produce compounds that are inhibitory to *Af* biofilm and that the inhibition by *Pa* is not unique to our reference strain of *Af*.

### The effect of *Pa* culture filtrates on *Af* is a result of inhibitors in filtrates, not reduction of nutrients

**Fig 7:**
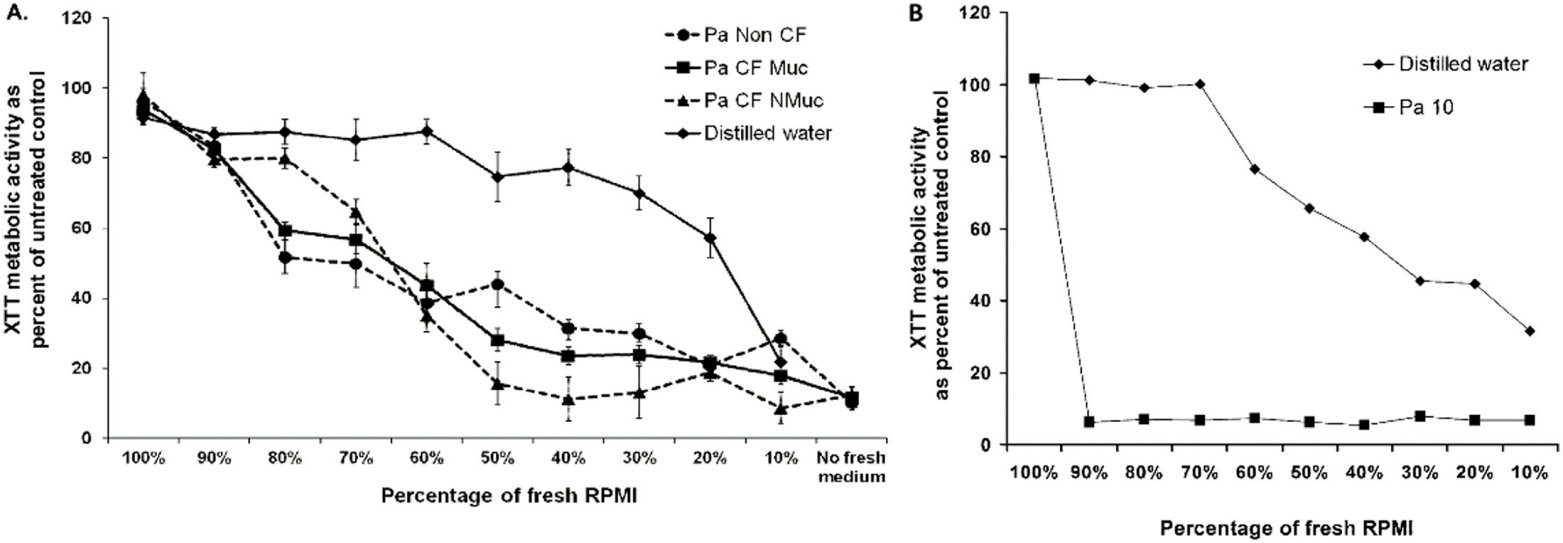
** A. *Pa* biofilm supernatant inhibits *Af* biofilm formation.** *Af* conidia were exposed to serial dilutions of *Pa* spent medium or sterile distilled water, mixed with fresh RPMI supplemented with 10% serum during *Af* biofilm formation as described in Methods and Fig 1III. The percent of fresh medium in each test situation was decreased as the spent medium or sterile distilled water increased. Then the resulting XTT readings were quantified, and the results expressed as percent of control with medium alone (no supernatant or distilled water). Results are presented as the mean of three replicates from three strains of *Pa* performed on two separate occasions. Error bars represent the SD of the mean. **B. *Pa* biofilm supernatant inhibits planktonic *Af*.** *Af* conidia were exposed to serial dilutions of *Pa* spent medium or sterile distilled water, mixed with fresh RPMI, and grown planktonically in tubes, as described in Methods. The percent of fresh medium in each tube was decreased as the spent medium or sterile distilled water increased. Then the resulting XTT readings were quantified, and the results expressed as percent of control with medium alone (no supernatant or distilled water). The results shown are with the non-mucoid CF *Pa* isolate supernatant; the mucoid CF *Pa* isolate and the non-CF *Pa* isolate were studied concurrently, and the results were not different; only the non-mucoid *Pa* result is shown to avoid clutter.

We addressed the possibility that the inhibition of *Af* biofilm by the *Pa* filtrates would be due to a reduced nutritional value of the spent culture filtrates diluting out the value of newly added medium during the incubation phase in the *Af* biofilm assays. The effect of fresh RPMI diluted with *Pa* culture filtrates (from biofilm-grown *Pa*) or sterile distilled water (a milieu that offers no nutrient support) on *Af* biofilm formation was studied. Fungal biofilm metabolic activity was inhibited over a range of ratios of fresh RPMI diluted with culture filtrates obtained from the representative CF and non CF *Pa* isolates, with inhibition starting at as little as 20% filtrate (80% fresh RPMI) (*P*<0.05) (Fig 7A). In contrast, *Af* biofilms were not significantly affected after challenge with fresh RPMI diluted in distilled water until the mixture contained only a 20% fresh RPMI concentration (80% distilled water) (Fig 7A); this concentration of RPMI is much lower than that of the 50% fresh RPMI dilution with filtrates as used in the supernatant experiments previously described (Figs 2 and 3). These findings indicate that the presence of inhibitory products in *Pa* culture filtrates were responsible for the inhibition of the metabolic activity of the fungal biofilms, rather than the inhibition being due to dilution of the nutrients in media by addition of *Pa* culture filtrate, or removal of the nutrients from the growth media during the *Pa* growth; i.e., that the inhibition by culture filtrate of *Pa* is a result of substances (<30,000 MW) *Pa* secretes into the culture filtrate, rather than nutrients removed by *Pa*.

Although this paper is focused on *Af* biofilm, it was of interest, for comparison, to see the effect of *Pa* supernatants on *Af* growing planktonically, as would occur in tubes, as in classical susceptibility testing [52]. The representative isolates from each of the three *Pa* groups was studied. When *Pa* supernatant was diluted in medium 1:1, exactly as in the challenges of biofilm, applied to *Af* conidia inocula in tubes, and the tubes examined when hyphal growth in matched controls was 4+, visible *Af* growth in the presence of supernatants did not occur (i.e., 0 growth in the supernatants of the CF isolates, or trace growth in that of the non-CF *Pa*). To again assure the inhibition seen was a result of toxic factors from the *Pa*, rather than depletion of media by *Pa* growth, the dilution series experiment with distilled water was repeated as above (Fig 7B). The result was even more dramatic than seen with *Af* biofilm targets: any concentration of supernatant ≥10% (i.e., ≤90% fresh medium) reduced the planktonic growth metabolic activity by XTT virtually completely, whereas it was only when the concentration of distilled water reached 40% (only 60% fresh medium) that any effect at all was seen on that XTT result (Fig 7B). The effect was modestly larger with the non-mucoid CF *Pa*, but was not statistically significantly greater than the inhibition seen with the supernatants of the other two *Pa* isolates.

### Effect of temperature on *Pa* supernatants

Two experiments were performed on heat treatment of the *Pa* filtrates (from planktonic or biofilm cultures), using the 3 representative isolates detailed, and (a) in the absence of serum from all steps or (b) just during the generation of supernatants. Comparison was made to untreated filtrates.

The heat treatment of the supernatants of all 3 *Pa* isolates, under both test conditions “(a)” and “(b)”, removed all inhibitory activity against *Af* forming biofilm or preformed *Af* biofilm, i.e., heated supernatant results same as controls (data not shown). There was one small exception: the non-mucoid CF strain heated supernatant retained significant inhibitory activity against *Af* forming biofilm, but not against preformed *Af* biofilm, only when the filtrate was from the *Pa* biofilm (not planktonic *Pa*) culture, and only in condition “(b)”.

In another experiment, freeze-thawed supernatant of the non-mucoid CF isolate, either grown planktonically or as biofilm, retained inhibitory activity against *Af* forming biofilm (both *P*<0.001 compared to untreated *Af* control), but this was significantly diminished compared to untreated supernatants (both *P*<0.001). When either frozen-thawed supernatant was tested against preformed *Af* biofilm, all activity was lost.

We conclude that the heat treatment used removed essentially all *Af*-inhibitory activity from supernatants of all types of *Pa* isolates. Freeze-thawing significantly diminishes inhibitory activity.

### Effect of DNase I and proteinase K on *Pa* biofilm culture filtrate activity

To determine whether the inhibitory substances were protein or DNA, we treated a nonmucoid *Pa* culture filtrate with DNAase or proteinase prior to assay for inhibitory activity. Compared to untreated *Pa* filtrate, filtrate treated with DNase I showed no significant differences in inhibitory capacity compared to *Af* control, nor did the DNase I alone have an inhibitory effect (data not shown).

EDTA (a chelator of metal anions, such as Fe; and a component of the DNase assay) alone reduced the metabolic activity of *Af* biofilm compared to *Af* biofilm untreated controls (*P*<0.001). The EDTA inhibitory effect was less than that of untreated *Pa* filtrate. EDTA + DNase I did not affect inhibition of *Af* biofilm by *Pa* filtrate. Finally, DNase I had no effect on EDTA inhibition of Af biofilm, as there were no significant differences between EDTA and DNase I + EDTA.

Proteinase K alone did not affect Af biofilm formation. The addition of Proteinase K to *Pa* filtrates did not affect filtrate inhibition of *Af* biofilm formation.

### Other characterization of *Pa* isolates

Because of the differences we observed among the *Pa* types tested, we also tested for some phenotypes previously associated with *Pa* virulence. Elastase activity, a virulence factor in *Pa* [57], was assessed in the *Pa* isolates that were used in the *Af* biofilm studies. The 5 non-mucoid CF isolates more frequently showed a positive test compared to the 5 mucoid CF and 5 non-CF isolates (3 vs. 2 and 1, respectively, at 24 hrs.; 5 vs. 3 and 3 at 48 hrs. with additional incubation at 4°C).

Certain *Pa* colonial types have been associated with *lasR* gene (a “master” gene controlling Pa virulence genes) mutations, more severe lung disease and persistence despite eradication attempts, including a wrinkly colony surface, irregular colony edge, metallic sheen, and green colony color [58]. As with elastase, these characteristics were also not significantly associated with our three groups differing in *Af* inhibition; 2 or 3 (of 5) of the non-mucoid isolates had each of these characteristics, compared to 1 to 3 of the other groups of 5 in each instance.

### Role of iron in inhibition by Pa filtrates

**Fig 8:**
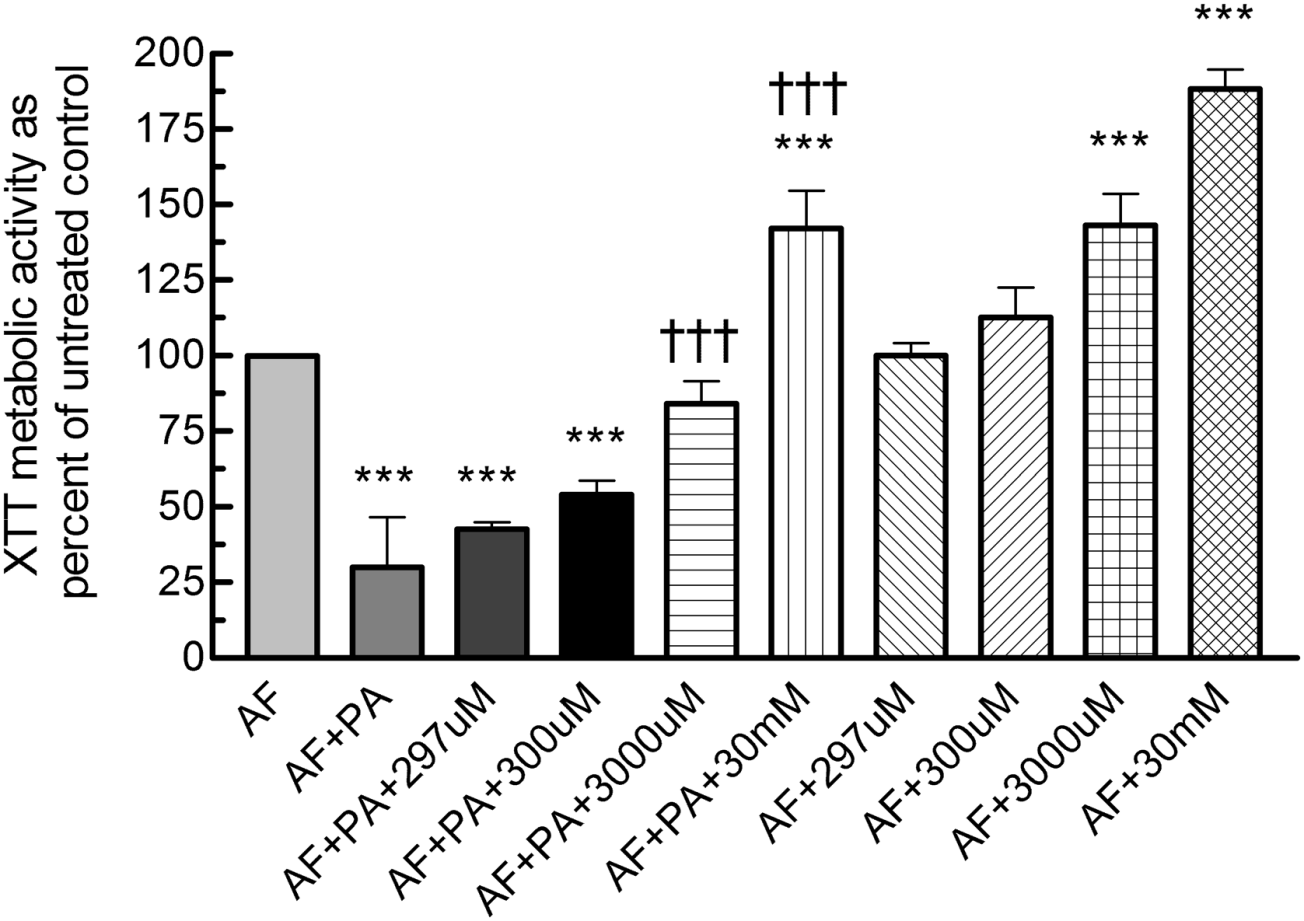
The effect of FeCl_3_ on the inhibition of *Af* biofilm formation, by filtrate from a CF non-mucoid *Pa* grown under biofilm conditions. Conidia formed biofilms on polystyrene disks (AF) during 16 h exposure to *Pa* filtrate (PA), filtrate + varying concentrations of FeCl_3_ (shown as the concentration, uM = μM), or varying concentrations of FeCl_3_ only. After 16 h of challenge, disks were transferred to fresh RPMI-1640 for 24 h of further growth before biofilm formation was quantified via XTT assay, and the results expressed as percent of control with medium alone (no supernatant or FeCl_3_). Asterisks denote *P* <0.001 for comparisons to the experimental control containing *Af* with RPMI-1640 (AF; no test materials). Daggers denote *P* <0.001 for comparisons with the experimental control containing *Af* +filtrate (AF + PA). Results with AF + PA + 11, 33 or 99 μM FeCl_3_ were not different than AF + PA; AF + 11, 33, or 99 μM FeCl_3_ were not different than AF + 297 μM FeCl_3_; and these results are not shown.

Iron is a co-factor required for growth and both *Af* and *Pa* produce siderophores to acquire iron for their needs. Three experiments were performed to confirm a possible role for iron sequestration by *Pa* filtrates in the inhibition of *Af*. A filtrate of the inhibitory non-mucoid *Pa*, grown under biofilm conditions, was tested (schema as in Fig 1V) alone or with added FeCl_3_, at 3-fold increasing concentrations from 11 μM through 297 μM, then 10-fold increasing concentrations from 300 μM through 30 mM, against Af forming biofilm, and compared to the usual control (RPMI without filtrate) (Fig 8). In combinations with filtrate, from 11 to 300 μM FeCl_3_, inhibition by the filtrate was retained, and without significant difference from the inhibition by filtrate in the absence of FeCl_3_ (*P*<0.001 for inhibition by all, compared to no-filtrate control). At 3000 μM FeCl_3_, there was no significant inhibition compared to control *Af*, and the inhibition was significantly less (*P*<0.001) than that with the filtrate without FeCl_3_. At 30 mM FeCl_3_ the XTT result showed not only lack of inhibition, but was significantly *enhanced* compared to both the filtrate-free control and filtrate without added FeCl_3_.

In separate studies on FeCl_3_ alone, reported preliminarily elsewhere [59], we have found, as in Fig 8, FeCl_3_ alone at ≥2500 μM to enhance *Af* biofilm XTT activity. The enhancement of growth in the present study, with 3000 μM FeCl_3_ alone, compared to partial inhibition by the same concentration combined with filtrate, confirms the dose-responsive reversal of inhibition by filtrate shown in Fig 8.

We conclude, from the iron reversal of *Pa* supernatant inhibition, that iron sequestration, such as in the form of *Pa* siderophores or other *Pa* iron-binding molecules [60], is at least part of the mechanism of the inhibition of *Af* biofilm by *Pa* filtrates.

## Discussion

Whereas planktonic *Af* growth appears affected by any *Pa* isolate, and the inhibition is more intense than on *Af* biofilm growth, we show that the described inhibition of *Af* biofilm by *Pa* [42–45] is related to the source and phenotype of the *Pa* isolate. Titration studies may have elucidated differences, among *Pa* types, in inhibition by supernatants on *planktonic Af* growth, but we chose not to pursue this. The differential effects of *Pa* types on *Af* biofilm is consistent with the extensive phenotypic and genomic changes in the mutable *Pa* organism that, associated with chronic residence in CF airways, have been described [2,8,9,61]. Specifically, we show live *Pa* cells inhibit the growth and formation of *Af* biofilm, with CF *Pa* isolates more inhibitory, and non-mucoid CF *Pa* isolates most inhibitory. Inhibition did not require the presence of *Pa* cells, as *Pa* culture filtrates were also inhibitory. The inhibition by filtrates showed the same hierarchy, with CF *Pa* isolates more inhibitory, and non-mucoid *Pa* isolates most inhibitory. Preformed *Af* biofilm was more resistant to *Pa*; non-mucoid CF *Pa* isolates were most inhibitory, and, again, that inhibition could be reproduced with culture filtrates. Reproducibility of the three *Pa* groupings was assured by the repeated experiments of various conditions, utilizing representative isolates of the groupings, as well as the number of isolates studied. Moreover, selection of *Pa*19, the most inhibitory of the non-CF isolates, as the representative of that group, would have minimized the differences in these studies between the CF and non-CF isolates.

Inhibition of *Af* biofilm appears dependent on the bacterial growth conditions, since filtrates from *Pa* grown as biofilm were more inhibitory than from *Pa* grown planktonically. However, the relationship of the number of Pa cells at the end of biofilm growth vs. the number after planktonic growth is unknown. Differences in biological effect on mammalian cells for molecules produced by planktonic *Pa* vs. biofilm *Pa* have previously been described, although planktonic *Pa* in that study produced more such active molecules [19]. We have assumed the production of substances with different *Af*-inhibitory power by the three *Pa* groupings is unrelated to the growth of the *Pa*, based on our growth studies by representative isolates of each group. However, each of the groups studied may be heterogenous, and more extensive studies, with more members of each group, might then show a relationship between growth and supernatant inhibition.

We chose to measure *Af* inhibition by the XTT assay of metabolism (which does not exclude the possibility of death of some *Af* cells), because of the controversies in interpreting CFU reduction in a multinucleate filamentous organism, commonly with incomplete septa between cells [62,63]. Such problems are magnified when attempting to quantitate a biofilm network [64]. However, others have indicated *Af* is, in fact, killed by *Pa* [64]. The XTT results would also not exclude a switch by the fungus to non-oxidative, fermentative, metabolism. However, our photomicrographs, and measurements of biofilm thickness, support the concept of actual growth inhibition occurring. The effects we describe on preformed *Af* biofilm show that *Pa* effects on *Af* are not solely on inhibition of the initial adhesion stage of *Af* biofilm development.

Previous work has indicated many candidate *Pa* molecules that can explain the various inhibitions we have described [42–47]. We confirm that small *Pa*-derived molecules, probably not nucleic acids or proteins per our studies, can explain at least most of the inhibition in supernatants. Whereas most previous work has focused on the toxic effects of *Pa* metabolites on fungi, our work suggests that denial of Fe to *Af* by *Pa* products is an important part of the inhibition. This is in contrast to a study of *Pa* inhibition of *Candida albicans*, which concluded that inhibition of that fungus was not a result of Fe limitation [46], although these apparent fungal differences could be affected by different experimental conditions. The bacterial-fungal interplay is more complex than just molecules produced by *Pa*, as recent elegant work has shown that *Af* can transform *Pa* metabolites, and thus radically alter the effect on the interaction, including the degree of inhibition [65]. Finally, Fe limitation by *Pa* could also in part be a result of modulation of *Af* siderophore production by *Pa* metabolites [66].

*Pa* quorum-sensing systems control expression of virulence factors, such as those previously implicated in *Af* inhibition [42–47]. A study that indicated no difference in quorum-sensing between CF and non-CF *Pa* isolates [67] might suggest that quorum sensing-induced molecules are at least not solely responsible for the increased inhibitory activity we have described for CF non-mucoid isolates. Some phenotypic factors previously associated with *Pa* virulence [57,58] that we studied did not appear associated with *Af* inhibition.

Our findings contrast with others, who reported *Af* biofilm was not inhibited by *Pa* [45], a difference likely attributable to their lack of study of CF *Pa* isolates. Our findings also contrast with the report of heat-resistance of *Af*-inhibitory factors produced in *Pa* cultures [45], since we show lability of essentially all the inhibition to heat, as well as significant lability to a freeze-thaw cycle. Such study differences could be a result of different *Pa* and *Af* culture and/or heating conditions. These differences support the value of detailing the physical and chemical characterizations of the supernatants described here; these characterizations could lead to further identification of the molecules responsible for the inhibition we have described. Another pathway to this end would be to utilize the many *Pa* virulence factor mutants [68] that have been described (studies in progress).

A limitation of our studies is that we have only studied one time period, although one adequate for bacterial growth, for production of *Pa* factors, whereas different regulatory systems may have different kinetics [68]. It may be of interest to study earlier and later time periods of *Pa* culture, and assess whether inhibitory factors are produced to a greater or lesser extent. We also cannot state at this time whether the enhanced inhibitory activity by biofilm *Pa* compared to planktonic *Pa* culture filtrates, or that resulting from non-mucoid CF isolates and their filtrates, is owing to increased production of inhibitory factors, or possibly the production of different factors. Nor is it known whether the inhibition by live cells relies solely on the same inhibitory factors (factors which can be released by *Pa* cells in close proximity to *Af* without dilution in culture medium) as in supernatants or whether there are unique factors resulting from or triggered by microbial contact. In this regard, the apparent greater inhibitory activity of live cells compared to supernatants (e.g., Fig 2) could be related to the observation that *Pa* production of virulence factors can be increased by co-culture with some other microbes [69]. It would also be of interest to study prolonged mixed *Pa*-fungal biofilm development [64], since the mixed condition is only beginning in our studies of *Af* forming biofilm challenged with *Pa* cells. Another issue is that CF patients in proximity to each other can cross-infect *Pa* isolates [70]; moreover, one *Pa* clone can therefore come to dominate in patients in one clinic-hospital setting [71]. Thus, without detailed sequencing data, one cannot be sure isolates from multiple CF patients in the same setting are not all very closely related; our observations should be confirmed with isolates from other centers. Finally, a population study, similar to that which we have done for *Pa*, would be of interest with respect to CF *Af* isolates, and whether any differ from non-CF, or reference *Af*, isolates (studies in progress).

Individual patient samples in our cohort shows not only that *Pa* isolates of varying colonial phenotype can co-exist in the same patient, but isolates with varying *Af*-inhibitory properties can co-exist; such polymorphism could be a transition state in the evolution of *Pa* colonization. Overall, from our studies of different *Pa* phenotypes, we hypothesize the transition of *Pa* from non-mucoid to mucoid [72–75], a process that usually occurs with time in CF disease, with the latter moving to a deeper zone of the lung [75], ablates *Pa*’s inhibitory effect on *Af* biofilm, and thus, helps explain why establishment of *Af* in the airways usually occurs later in CF disease. Evolution of *Pa* may act in concert with other factors favoring *Af* colonization [76], including repeated antibacterial courses.

## Supporting Information

S1 Table Compendium of studies reported. (DOCX)
