# Supplementary material for: Inhibition of Aspergillus fumigatus and Its Biofilm by Pseudomonas aeruginosa Is Dependent on the Source, Phenotype and Growth Conditions of the Bacterium
Source: PLoS One. 2015 Aug 7;10(8):e0134692. doi: 10.1371/journal.pone.0134692 (PMC4529298; doi:10.1371/journal.pone.0134692)
Supplement: S1 Table — (DOCX) [file pone.0134692.s001.docx]

***Supplementary table***

**Compendium of studies reported**

1. Af biofilm formation as affected by live cells of: non-CF Pa

mucoid CF Pa

nonmucoid CF Pa

2. Af biofilm formation as affected by filtrates of: Pa’s grown planktonically (each of the 3 Pa types above)

Pa’s grown as biofilm (each of the 3 Pa types above)

3. Af preformed biofilm as affected by live cells of: non-CF Pa

mucoid CF Pa

nonmucoid CF Pa

4. Af preformed biofilm as affected by filtrates of: Pa’s grown planktonically (each of the 3 Pa types above)

Pa’s grown as biofilm (each of the 3 Pa types above)

5. Effect of Pa growth on Af-inhibitory power of filtrates

6. Effect of serum on Af-inhibitory power of filtrates

7. Comparison of filtrates grown planktonically or as biofilm from the same isolates

8. Confocal analysis of filtrates on Af biofilm formation or Af preformed biofilm

9. Molecular weight characterization of inhibitors

10. Specificity of inhibition

11. Inhibitors in filtrates compared to dilutions of filtrates, comparison on planktonic and biofilm Af growth

12. Effect of temperature on inhibition by Pa filtrates

13. Effect of DNase and proteinase on inhibition by Pa filtrates

14. Various phenotypic studies on Pa’s and correlation with Af inhibition

15. Role of Fe in inhibition by Pa filtrates.
